# In the midst of the unending risk of disease, how can sub-Saharan Africa accelerate progress on its industrial development?

**DOI:** 10.11604/pamj.2024.49.64.45120

**Published:** 2024-11-06

**Authors:** Rexford Kweku Asiama

**Affiliations:** 1 University of Johannesburg, Johannesburg, South Africa,; 2 University of Environment and Sustainable Development, Somanya, Ghana

**Keywords:** Disease, industrial, development, COVID-19

## Abstract

Despite a high disease burden, sub-Saharan Africa's mortality rates from diseases are currently lower than those in other regions, largely due to the continent's younger population. However, the region is more susceptible to health risks, resulting in frequent health emergencies. Even recently, monkeypox, which originated in an African country, has been declared a global health emergency. Sub-Saharan Africa´s predominantly youthful demographic presents a valuable opportunity to use this advantage to accelerate progress toward sustainable industrialization. By analyzing pre- and post-pandemic trends in major sectoral indicators, population dynamics, and technology exports, this discussion explores the potential for sub-Saharan Africa to enhance its long-term industrial growth.

## Commentary

Africa carries the world´s largest disease burden estimated at about 20% of the global burden [1], with the most recent havoc caused by the coronavirus disease (COVID-19) pandemic. The World Health Organization (WHO) declared COVID-19 a public health emergency of international concern, soon after Africa reported its first case on February 14^th^, in Egypt, and the virus was classified as a global pandemic on March 12^th^. By May, COVID-19 had reached every country in Africa, predominantly entering capital cities via international flights from Europe and subsequently spreading through community transmission.

Community transmission of the pandemic spread swiftly in some of Africa's major countries, as expected given the amount of urbanization, such as South Africa, which has the most cases, despite Egypt having the most COVID-19 deaths in Africa. Algeria, Morocco, Nigeria, and Ghana are among the countries that have suffered significantly [2,3]. Africa's mortality rates are currently far lower than elsewhere, owing to the continent's youthful population [4]. While the accuracy of testing and case data in Africa is called into question, the pandemic and other health disasters are more likely to occur in sub-Saharan Africa, relative to other regions. Aside from the pandemic, there has been recent news of monkeypox, which has been recognized as a global health emergency and originated in an African country [5].

However, with a predominantly young population, sub-Saharan Africa has the ability to find a unique opportunity that capitalizes on the health and well-being of its younger population in order to speed progress towards sustainable industrialization [4]. In this regard, this draft examines the trends in main sectoral value addition, technology exports, and the youthful population in sub-Saharan Africa, as well as the prospects for the region to accelerate its progress toward industrialization. The subsequent parts discuss the importance of industrialization, trends in various crucial metrics, and the potential for the region before providing the major conclusions.

### Why should industrialization still matter?

Countries and regions around the world vary significantly in their levels of development. Some nations today are wealthier and enjoy higher standards of living compared to others. Over the years, development economists have explored various theories to explain why some countries are more advanced than others. Among these, the structuralist perspective is particularly relevant to sub-Saharan Africa. Structuralists argue that economic growth is driven by the contributions of specific sectors within an economy [6,7]. According to this view, the way different sectors produce value-added goods and services plays a crucial role in driving economic growth. As a result, the productivity of certain sectors relative to others can be a key driver of overall growth. This structuralist approach is especially important for sub-Saharan African countries, many of which are still at lower levels of development.

### A review of key industrial and population trends in SSA

**Figure 1:**
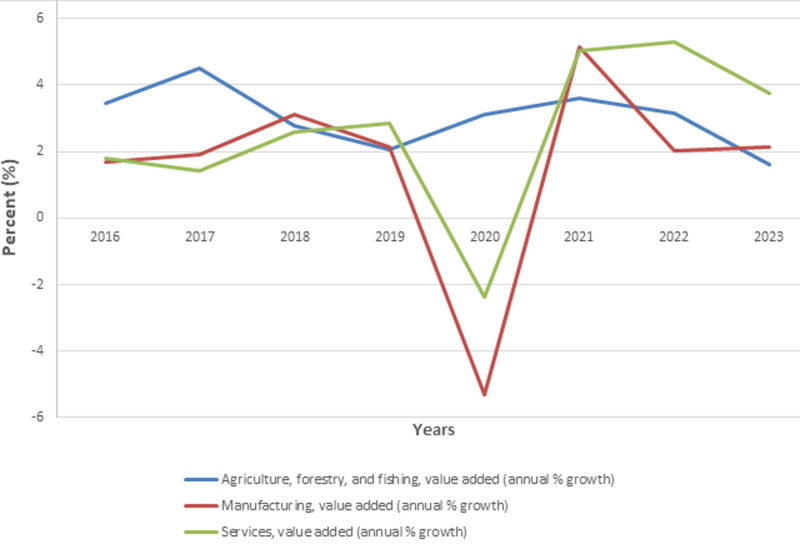
pre- and post-pandemic sectoral value-added trends in sub-Saharan Africa (source: World Development Indicators)

The economic performance of sub-Saharan Africa, excluding high-income countries, has shown notable sectoral fluctuations over recent years (Figure 1). The agricultural sector, which is vital to the livelihoods of many in the region, experienced a peak in growth at 5% in 2017. However, subsequent years saw a decline, with the growth rate decreasing to 2% by 2023. This downward trend indicates potential challenges in agricultural productivity and resilience, necessitating further investigation into the factors that may be inhibiting sustained growth in this sector.

The manufacturing sector has displayed significant volatility, particularly with a sharp contraction of 5% in 2020 (Figure 1). This decline was likely influenced by global disruptions, such as the COVID-19 pandemic, which severely impacted industrial activity. However, the sector rebounded robustly in 2021 with a 5% growth rate, reflecting a temporary recovery. Despite this rebound, the growth rate in manufacturing stabilized at a modest 2% in the following years, highlighting ongoing uncertainties and the need for policies that bolster industrial resilience.

In contrast, the services sector has demonstrated a steady recovery after a contraction of -2% in 2020 (Figure 1). This sector grew consistently, reaching a peak growth rate of 5% in both 2021 and 2022. Although there was a slight decline to 4% in 2023, the services sector's overall performance underscores its importance as a driver of economic activity in the region. The sector's resilience, even in the face of external shocks, suggests a strong foundation that could be further leveraged for economic stability.

**Figure 2:**
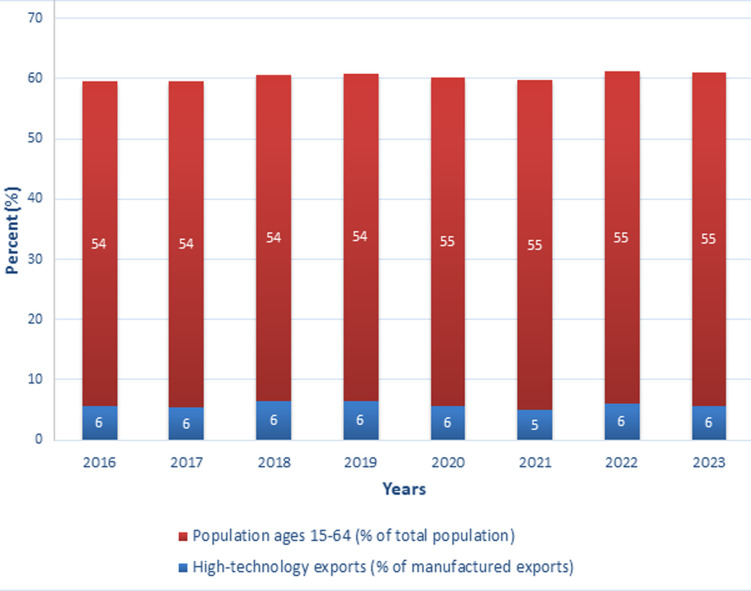
pre- and post-pandemic trends in high-technology exports and youthful population in sub-Saharan Africa (source: World Development Indicators)

High-technology exports, as a percentage of manufactured exports, have remained relatively stable at 6% over the years, with a minor dip to 5% in 2021 (Figure 2). This consistency points to a sector that has not seen significant growth but also has not contracted. The stagnation in this area suggests that there may be untapped potential for innovation and expansion in high-technology industries, which could be crucial for diversifying the region's economic base and enhancing its global competitiveness.

The population of working-age individuals (ages 15-64) in sub-Saharan Africa has remained stable at around 54% from 2016 to 2019, with a slight increase to 55% starting in 2020 (Figure 2). This demographic stability, coupled with a gradual increase, indicates a growing labor force that could be leveraged for economic growth. However, the slow pace of this demographic shift may also reflect underlying issues in health, education, and employment opportunities that need to be addressed to fully capitalize on the region's human capital.

Overall, the data presents a mixed picture of economic performance in sub-Saharan Africa, with certain sectors like services showing resilience, while others, such as agriculture and manufacturing, reveal structural vulnerabilities that could affect the long-term development of the continent. Nonetheless, the stability in high-technology exports and the gradual demographic shift in the working-age population suggest areas for potential growth and development.

### The ´blessings´ of COVID-19 and the new opportunities for African industrialization

COVID-19 had the potential to boost Africa´s information technology sector in a manner similar to how the Y2K crisis benefited India. India capitalized on the global crisis by leveraging its large youth population to train millions of software engineers, who now lead multinational companies worldwide. Similarly, Africa could harness the pandemic´s impetus to enhance its tech industry. The pandemic accelerated global digital technology adoption, making virtual connections essential, and highlighted significant shortages of technology talent, especially in Africa where economic and policy developments have been slower to keep pace [8].

The African Union and its member countries are eager to boost industrialization, but the current policy framework presents significant hurdles. Existing policies frequently prioritize overall investment climate challenges over targeted support for individual sectors. Furthermore, even if there is a commitment to industrial strategy, weak institutions or unfavorable political conditions can impede implementation. The COVID-19 pandemic has increased skepticism about Africa's ability to rapidly industrialize in a complex global setting. However, the crisis has also highlighted three positive developments critical to economic recovery: the repurposing and acceleration of pharmaceutical production and collaborative procurement, a greater emphasis on agro-processing, and the adoption of technical improvements. Beyond these immediate concerns, Africa should emphasize green industrialization, digitalization, and regional integration to enhance its role in global industrialization efforts over the coming decade [9].

### Conclusion - an unending cycle?

The inadequate health systems seen in many African countries pose a severe danger to the stability of economic activity in sub-Saharan Africa, with the potential to undermine progress towards industrialization and larger development goals. The recent monkeypox outbreak, which has been formally recognized as a global health emergency, as well as the appearance of other associated fevers, emphasizes the urgent need for significant improvements in healthcare delivery across the continent. The situation serves as a clear reminder that, while some progress has been made, far more work remains to be done to establish resilient health systems capable of efficiently addressing crises.

A well-functioning healthcare system is not just a necessity for addressing immediate health challenges; it is also a foundational element for sustainable economic growth. A healthy workforce is essential for driving industrial development, and the absence of robust healthcare services can severely undermine efforts to mobilize the region's young and dynamic population. By improving health outcomes, African countries can enhance the productivity of their labor force, which is critical for accelerating industrialization and unlocking the full potential of the region's demographic dividend. Investing in health is, therefore, not just a social imperative but also an economic one, crucial for the long-term development and prosperity of sub-Saharan Africa. Furthermore, strategic interventions aimed at enhancing productivity, fostering innovation, and addressing demographic challenges could be key to sustaining and accelerating economic progress in the region.
